# Effect of Apheresis for ABO and HLA Desensitization on Anti-Measles Antibody Titers in Renal Transplantation

**DOI:** 10.1155/2011/869065

**Published:** 2011-12-08

**Authors:** Ulf Schönermarck, Teresa Kauke, Gundula Jäger, Antje Habicht, Thorsten Wendler, Joachim Andrassy, Markus Guba, Manfred Stangl, Michael Fischereder

**Affiliations:** ^1^Nephrology Division, Department I of Internal Medicine, University Hospital Munich—Campus Grosshadern, Ludwig-Maximilians-University Munich, Marchioninistrasse 15, D-81377 Munich, Germany; ^2^Laboratory of Immunogenetics, University Hospital Munich—Grosshadern, Ludwig-Maximilians-University, Munich, Germany; ^3^Department of Surgery, University Hospital Munich—Grosshadern, Ludwig-Maximilians-University, Munich, Germany; ^4^Department of Clinical Virology, Max von Pettenkofer-Institute, Ludwig-Maximilians-University, Munich, Germany; ^5^Transplant Centre, University Hospital Munich—Grosshadern, Ludwig-Maximilians-University, Munich, Germany

## Abstract

Desensitization strategies for ABO-incompatible renal transplants with plasma exchange (PE) or specific immunoadsorption (IA) decrease immunoglobulin levels. After recent measles outbreak and decreasing vaccination rates, we studied the impact of apheresis on anti-measles antibodies. Anti-measles antibodies were measured before desensitization, before transplantation and during followup in 12 patients with ABO incompatibility (2x PE only, 8x IA only, and 2x IA and PE) and 3 patients with donor-specific HLA antibodies (all PE). Patients received rituximab, IVIG, and standard immunosuppressive therapy. All patients had detectable anti-measles antibodies before desensitization (mean 3238 mU/l, range 560–8100). After 3–6 PE sessions, titers decreased significantly to 1710 mU/l (*P* < 0.05), in one patient to nondetectable values, while IA only maintained protective titers. After a median followup of 64 days, anti-measles antibodies returned to baseline in all patients. Immunity against measles was temporarily reduced by apheresis but remained detectable in most patients at time of transplantation. Desensitization maintains long-term protective immunity against measles.

## 1. Introduction

Desensitization protocols have been successfully implemented worldwide for removal of donor-specific blood group or HLA antibodies in the recipient to allow transplantations across blood group and HLA barriers. Although there is a high variability within these protocols, results for ABO-incompatible transplantations are comparable to those used for ABO-compatible transplantations regarding patient and graft survival worldwide [1–8].

Current therapeutic apheresis techniques to remove blood group antibodies include plasma exchange, double-filtration apheresis, protein A immunoadsorption, and antigen-specific immunoadsorption (Glycosorb). Except for the latter, these techniques are also suitable for removal of HLA antibodies [9]. More selective methods have the advantage that large plasma volumes can be processed with increased efficacy, but without removal of coagulation factors and need for replacement fluids. However, other donor-specific antibodies, such as HLA antibodies, are not removed, and removal of complement factors may offer additional advantages. Both can be achieved by “unspecific” plasma exchange, which is the most common technique used worldwide due to availability.

With decreasing specificity, these regimens also result in increasing removal of protective immunoglobulins resulting from previous diseases or specific vaccinations. At present, there is limited evidence that immunity to vaccinations with antigens which readily elicit an immune response such as tetanus toxoid is maintained after any of the above desensitization protocols. However, the effects on immune responses from vaccines with weaker antigens are far less clear. In this respect, especially the protective effects from vaccinations with live vaccines appear of interest as such vaccinations are contraindicated after solid organ transplantation. Furthermore, recent outbreaks of measles in Germany have raised the question, if a decreasing performance of vaccination and lower herd immunity in the population pose a risk for measles infection in immunocompromised patients after kidney transplantation, especially in view of new and more intensive treatment strategies using apheresis techniques. Therefore, we studied the effect of various desensitization protocols on anti-measles antibodies.

## 2. Materials and Methods

### 2.1. Patients

Between March 2007 and April 2011, at our kidney transplant center, 17 patients were successfully transplanted after desensitization for ABO or HLA incompatibility. Two patients had to be excluded from this analysis because of incomplete data. All patients gave informed consent for participation in the study.

### 2.2. ABO-Incompatible Transplantation

All 12 patients with ABO incompatibility received living donor kidney transplants and had a negative complement-dependent cytotoxic T-cell crossmatch. The desensitization protocol was adopted according to previously published work [7, 8]. Briefly, rituximab (375 mg/m²) was given ~4 weeks before the anticipated transplantation. Triple oral immunosuppression with tacrolimus, mycophenolate mofetil, and prednisolone or methylprednisolone was started with initiation of apheresis. Induction therapy was performed with basiliximab (day of surgery and postoperative day 4) or antithymocyte globulin (1 to 3 doses). Intravenous immunoglobulin (0.5 g/kg) was only given in the first and second patients 4 days before surgery and thereafter omitted from the protocol. CMV prophylaxis with CMVIg was only given in the first patient postoperatively. All other patients received antiviral prophylaxis with valganciclovir and cotrimoxazole for 3 to 6 months.

For removal of blood group antibodies, plasma exchange or antigen-specific immunoadsorption was used. During each plasma exchange, one-plasma volume was replaced with 5 percent serum albumin, on the day of surgery or immediately after surgery replacement consisting of 5 percent human albumin and one liter of fresh frozen plasma. Antigen-specific immunoadsorption was performed using an antigen-specific carbohydrate column (Glycosorb A/B, Glycorex Transplantation AB, Sweden). During each session, 2-3 plasma volumes were processed. In two patients only plasma exchange and in eight patients only antigen-specific immunoadsorption was performed. In another two patients, isoagglutinin titer reduction was not successful using antigen-specific immunoadsorption, and therefore plasma exchange was added. Apheresis was performed until anti-A/B IgG isoagglutinin titers were 1 : 4 or less on the day of surgery. Postoperative apheresis was only performed when the anti-A/B IgG isoagglutinin titers exceeded 1 : 8 in the first week and 1 : 16 in the second week after surgery.

### 2.3. HLA-Incompatible Transplantation

Three patients received desensitization therapy because of the presence of donor-specific HLA antibodies in the presence of a negative complement-dependent cytotoxic T-cell crossmatch. In two patients, living donor kidney transplantation was performed, while in one patient cadaveric kidney transplantation was performed after reduction of HLA antibodies. Rituximab (375 mg/m²) was given 4 weeks before the anticipated transplantation or in the latter patient at time of transplantation. A single dose of intravenous immunoglobulin (1 g/kg) was applied one week after the rituximab, that is, before the start of plasma exchange. Triple oral immunosuppression with tacrolimus, mycophenolate mofetil, and prednisolone/methylprednisolone was started with initiation of apheresis. Induction therapy was performed with basiliximab (day of surgery and postoperative day 4) or antithymocyte globulin (2 doses). CMV prophylaxis with CMVIg was given in one patient postoperatively (patient 15). All patients received antiviral prophylaxis with valganciclovir and cotrimoxazole for 3 to 6 months.

Plasma exchange was performed with one-plasma volume replaced by 5 percent serum albumin or postoperatively using 5 percent human albumin and one liter of fresh frozen plasma as substitution. Detection of anti-HLA antibodies was performed by Luminex technology. Sera of patients were tested by Lifecodes LSA Class I/II (Genprobe, Transplant Diagnostics, Stamford, USA) single antigen bead assay to identify anti-HLA antibody specificity and intensity.

### 2.4. Measurement of Anti-Measles Antibodies

Quantitative measurement of anti-measles IgG antibodies was performed in stored serum or plasma samples by an enzyme immunoassay used in routine laboratory practice. Briefly, IgG antibodies specific for measles in the test sample bind to the antigen (permanent simian kidney cells infected with measles virus) or as control to noninfected cells and are incubated at 37° for 1 h. After completing 4 wash cycles, the conjugate (anti-human IgG/POD) is added and incubated for 1 h. After washing, the substrate is added for 30 min at room temperature protected from light. Measurement is performed at 450 nm after adding of the stopping solution. Evaluation is performed automatically in BEP III (Siemens, Germany). A titer >150 mU/l was defined positive. Anti-measles antibodies were measured in serum samples before rituximab treatment, the day before transplantation and during followup. In three patients, anti-measles antibodies were determined at the beginning and at the end of a single plasma exchange session. Furthermore, in two patients, anti-measles antibodies were determined serially over a period of *∼*3 years, respectively.

## 3. Results

### 3.1. Baseline Anti-Measles Antibody Titers

Baseline data and treatment modalities are reported in Table 1. All patients had detectable anti-measles antibodies (>150 mU/l) before starting desensitization therapy (mean 3238 mU/l, median 2600 mU/l, and range 560–8100 mU/l). In two patients, anti-measles antibodies were tested serially before and after transplantation, in patient 6 six times over a period of 36 months, and in patient 5 seven times over a period of 32 months. Anti-measles antibodies remained stable over this period with a standard deviation of 9% and 13%, respectively.

### 3.2. Effect of Apheresis on Antibody Titers

Overall, ABO blood group antibodies and HLA antibodies were effectively reduced during the desensitization protocol using either plasma exchange, antigen-specific immunoadsorption, or both treatments.

**Figure 1:**
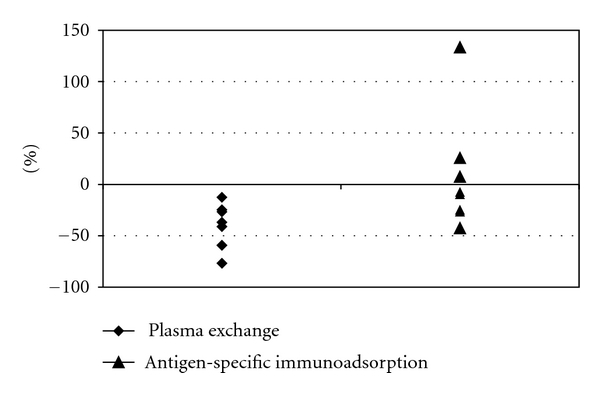
Effect of apheresis on reduction of anti-measles IgG antibodies. Antibody titer reduction (in %) at time of transplantation in comparison to the baseline values among patients using plasma exchange either alone or in combination with antigen-specific immunoadsorption (*n* = 7) or antigen-specific immunoadsorption alone (*n* = 8). There was a mean reduction of 40% in the plasma exchange group.

**Figure 2:**
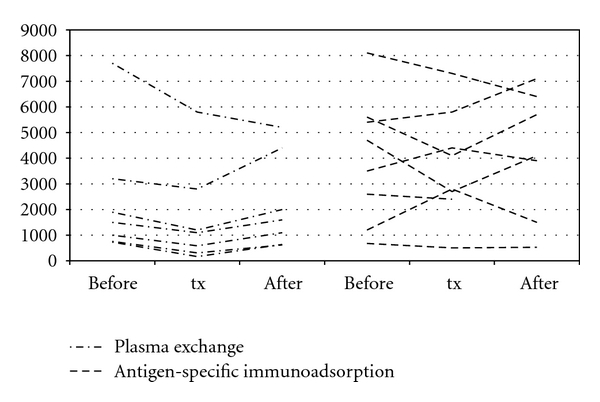
Effect of desensitization therapy on anti-measles IgG antibody titers (mU/l). Anti-measles antibodies were measured before desensitization therapy (“before”), the day before transplantation (“tx”) and during followup (“after”). Patients were analysed according to the used apheresis strategies: use of plasma exchange either alone or in combination with antigen-specific immunoadsorption (*n* = 7) or antigen-specific immunoadsorption alone (*n* = 8). At the time of transplantation, antibody titers were significantly reduced in the plasma exchange group compared to baseline (*P* < 0.05).

Anti-measles antibody levels were measured in 3 patients at the beginning and at the end of a single plasma exchange session. A single treatment reduced the antibody concentration by 42%. Using plasma exchange, anti-measles antibodies decreased significantly in all patients (*n* = 7), with reduction from a mean of 2399 mU/l (median 1500; range 730–7700 mU/l) to a mean of 1710 mU/l (median 1100; range 150–5800 mU/l; *P* < 0.05). This represented a decrease of 40% after 2 to 7 plasma exchanges (Figure 1). In two patients, anti-measles antibody titers were low at time of transplantation, and in one patient, the antibody concentration decreased to nondetectable values immediately after the plasma exchange session (Figure 2).

In patients using antigen-specific immunoadsorption, only (*n* = 8) anti-measles antibody levels decreased slightly, but without statistical significance (mean antibody level 3973 versus 3751 mU/l). All patients had highly protective antibody levels at time of transplantation (Figure 2).

### 3.3. Effect of Immunoglobulin Treatment

In four patients, intravenous immunoglobulins were given as part of the treatment protocol. In one patient treated with antigen-specific immunoadsorption, anti-measles antibody level increased at time of transplantation. The other three patients received intravenous immunoglobulins either one to two weeks before the start of plasma exchange (patient 14 and 15) or two days before the last plasma exchange (patient 2). Among patients using plasma exchange, anti-measles antibodies were lowered more effectively in those patients not receiving intravenous immunoglobulins, although due to the small number of patients without statistical significance (47% versus 29%, *P* > 0.1). There were no differences in clinical outcomes regarding infections between these patient groups.

### 3.4. Anti-Measles-Antibody Titers during Followup

During followup (median 64 days after transplantation, range 26–1114 days), all patients had protective immunity against measles (*n* = 14, median 2950 mU/l, and range 530–7100 mU/l). Anti-measles antibodies remained stable as compared to baseline values or increased in most patients after temporal reduction during desensitization therapy despite immunosuppressive therapy.

## 4. Discussion

A decrease of vaccination rates and a lower herd immunity have triggered recent outbreaks of measles in Germany and other countries. New and more intense treatment strategies utilizing apheresis techniques and increased immunosuppression might even further increase the infectious risk for transplanted patients. Furthermore, the possibility of atypical presentation of measles with nonspecific respiratory or neurological symptoms has to be considered in immunocompromised patients [10, 11].

Patients born before the implementation of vaccination against measles most likely had natural measles and lifelong immunity. However, children and young adults born after 1970 who are unvaccinated or have received only one dose of vaccine with suboptimal protection are at risk. In our population, all 15 adult patients, including 3 patients born after 1970, had protective levels of anti-measles antibodies prior to transplantation and desensitization therapy. In contrast, in children receiving solid organ transplants, a lower rate of protective antibody titers against measles (80%) and other viruses [12] and loss of antibodies to measles after transplantation in a significant proportion of individuals have been reported [13].

This limited degree of immunity against measles might be further reduced when potent desensitization protocols are used as, for example, in ABO-incompatible renal transplantation. The effect of therapeutic apheresis on specific antibody levels against bacterial antigens has been investigated only in small patient groups. Despite using antigen-specific immunoadsorption with Glycosorb A/B columns, several authors reported a significant reduction of total IgG and IgM [14, 15]. Antibodies against tetanus and diphtheria protein antigens were not significantly affected. However, antibodies against pneumococcus and haemophilus polysaccharide antigens were significantly reduced [14, 15]; in some patients, antibody levels decreased even below the protective threshold values.

We extend these findings by reporting the effect of plasma exchange and antigen-specific immunoadsorption on the levels of anti-measles antibodies. Plasma exchange significantly reduced anti-measles IgG titers, in 3/7 patients to low values, and in one patient even below the detection threshold immediately after the plasma exchange session. The effect of antigen-specific immunoadsorption was diverse and not statistically significant, although antibody levels decreased in several patients. The reduction of anti-measles antibody titers was merely transient, and anti-measles antibody levels remained stable as compared to baseline values or increased in most patients over time. However, it has to be considered that antibody reduction might be more pronounced in patients with preexisting low anti-measles-antibody titers and/or intensive use of apheresis techniques. In this setting, patients would be at high risk for posttransplant infection. Therefore, a pretransplant booster vaccination might be useful in patients with low titers.

In most protocols, intravenous immunoglobulins (IVIGs) or cytomegalovirus immune globulin (CMVIg) has been used as part of the desensitization therapy in combination with plasma exchange and antigen-specific immunoadsorption. Although the exact immunosuppressive mechanism is unknown, an immunomodulatory effect by replacing removed antibody and suppressing de novo antibody production is speculated [5]. However, preparation (CMVIg, several IVIG preparations), dosing, and timing are highly variable (0.1 g/kg after each plasma exchange; 2 g/kg after the final plasma exchange; 0.5 g/kg 1 or 5 days prior to transplantation using antigen-specific immunoadsorption) [1, 3, 4, 6, 7, 16, 17], and randomized trials are lacking. It should also be considered that different IVIG preparations contain blood group antibodies in variable titers [18]. Because of uncertainties about the mechanism and potential side effects [16], we and other groups have safely omitted routine application of IVIGs in ABO-incompatible transplantation.

Despite immunomodulatory effects, substitution with intravenous immunoglobulins (IVIG) can restore the protective IgG pool in the first weeks after transplantation, as has been shown previously [15]. IVIG and fresh frozen plasma contain a variety of protective antiviral antibodies including anti-measles antibodies in varying concentrations. As can be demonstrated in patient 1 using antigen-specific immunoadsorption, application of IVIG can increase the anti-measles antibody titer more than 100%. There was also a trend that among patients using plasma exchange, anti-measles antibodies were lowered less effectively in those patients receiving IVIG. However, in our study, IVIG was used as part of the desensitization protocol and not as substitution after completion of apheresis treatment. Regardless of the use of IVIG, anti-measles antibodies were significantly lowered using plasma exchange as compared to antigen-specific immunoadsorption. Fresh frozen plasma was only used in three patients immediately before or after transplantation. The impact on our results will likely be limited, as the use of one liter of fresh frozen plasma will not sufficiently and long lasting restore the IgG pool. With a half-life of ~20 days for IgG, the increase of the anti-measles antibody titers measured after transplantation (median 64 days) represents the endogenous production, rather than an effect of substitution with fresh frozen plasma or IVIG as used in our protocol.

Anti-measles antibody titers were lowered temporarily towards the detection limit only in those two patients using plasma exchange with low anti-measles antibody titers at start of desensitization therapy. However, in most patients, antibody levels remained above the protective threshold and substitution with IVIG will not be necessary.

## 5. Conclusions

Immunity against measles was detectable in all adult patients undergoing desensitization with plasmapheresis or immunoadsorption plus rituximab but was temporarily reduced at the time of transplantation. Anti-measles antibodies remained detectable in all but one patients at time of transplantation. Current desensitization does not compromise protective immunity against measles permanently.

## Figures and Tables

**Table 1:** Patient characteristics.

Patient	Age/Sex	IVIG	Blood group Donor→Recipient	Titer	Plasma exchange (before/after tx)	Immunoadsorption (before/after tx)
1	42 f	yes	A2 → B	1 : 4		4/0
2	45 f	yes	B → O	1 : 32	4/0	
3	17 m	no	A1 → O	1 : 32	3/1	4/0
4	23 m	no	A2 → O	1 : 64		3/0
5	66 f	no	A2 → O	1 : 8		2/0
6	64 f	no	A2 → O	1 : 8		2/0
7	41 m	no	A1 → O	1 : 1024	3/0	11/3
8	63 m	no	A1 → O	1 : 1024		9/1
9	64 m	no	A1 → O	1 : 256		8/0
10	51 m	no	B → A1	1 : 8		2/0
11	56 f	no	A1B → A1	1 : 128		4/0
12	48 f	no	A1B → A1	1 : 2	2/0	

			HLA antibodies			

13	47 m	no	HLA		11/9	
14	47 f	yes	HLA		6/2	
15	37 m	yes	HLA		7/0	

Age in years; sex: m: male, f: female. IVIG: intravenous immunoglobulin. Titer: in case of ABO-incompatible transplantation anti-A/B isoagglutinin titers before start of desensitization are given, and apheresis was performed until isoagglutinin titers were 1 : 4 or less on the day of surgery. The number of treatments using plasma exchange or antigen-specific immunoadsorption either before kidney transplantation (before-tx) or after successful kidney transplantation is given. Plasma exchange was usually performed with 5 percent albumin as replacement fluid. Additionally, fresh frozen plasma was used in patient 3 for the plasma exchange on the day before surgery and a plasma exchange 6 days after transplantation; in patient 14 for the plasma exchanges 2 and 4 days after transplantation; in patient 2 on the day of surgery.
